# Postpartum follow-up of women with preeclampsia: facilitators and barriers — A qualitative study

**DOI:** 10.1186/s12884-023-06146-8

**Published:** 2023-12-04

**Authors:** Eirin Arntzen, Ranveig Jøsendal, Heidi Linn Sandsæter, Julie Horn

**Affiliations:** 1 Department of Public Health and Nursing, Faculty of Medicine and Health Sciences, Norwegian University of Science and Technology, Postbox 8905, NO-7491 Trondheim, Norway; 2 Department of Obstetrics and Gynecology, Levanger Hospital, Nord-Trøndelag Hospital Trust, Levanger, Norway

**Keywords:** Cardiovascular disease prevention, Lifestyle change, Preeclampsia, Postpartum, Qualitative research

## Abstract

**Background:**

Pregnancy causes physiological changes to the maternal organ systems that can be regarded as a cardiometabolic stress test for women. Preeclampsia, a pregnancy complication characterized by new onset of hypertension in combination with proteinuria or end-organ dysfunction, affects approximately 2–8% of pregnancies. Adverse pregnancy outcomes, including preeclampsia, have been described as a failed stress test and have been consistently linked with increased risk of cardiovascular disease later in life. The postpartum period is therefore often regarded as a window of opportunity for cardiovascular disease prevention. However, we lack knowledge about how women with preeclampsia experience current postpartum care in the Norwegian health system. The aim of this qualitative study is to uncover women’s perspectives and preferences regarding postpartum follow-up.

**Methods:**

Semi-structured telephone interviews were conducted with 17 women following a six-month lifestyle intervention study. Participants were 9–20 months postpartum, following a pregnancy complicated by preeclampsia. Data were analyzed using Malterud’s systematic text condensation.

**Results:**

We identified five themes, each with 2–3 subthemes, that demonstrate how women with recent preeclampsia experience postpartum follow-up: (1) fear and uncertainty (a body out of balance and facing an uncertain future), (2) a conversation on lifestyle – not really that difficult (preeclampsia as a gateway, a respectful approach, and a desire for more constructive feedback), (3) when your own health is not a priority (a new everyday life, out of focus, and lack of support), (4) motivation for lifestyle changes (an eye opener, lack of intrinsic motivation, and a helping hand), and (5) lack of structured and organized follow-up (there should be a proper system, a one-sided follow-up care, and individual variation in follow-up care).

**Conclusions:**

Findings from this study highlight the need for more systematic postpartum follow-up for women after a pregnancy complicated by preeclampsia. Further research is required to explore the potential use of standardized guidelines and routine invitations to postpartum care. Furthermore, exploring health care professionals’ experiences is crucial to ensure their engagement in postpartum care after complicated pregnancies.

**Supplementary Information:**

The online version contains supplementary material available at 10.1186/s12884-023-06146-8.

## Background

Pregnancy causes physiological changes to the maternal organ systems that can be regarded as a cardiometabolic stress test for women [1]. Adverse pregnancy outcomes have been described as a failed stress test and have been consistently linked with increased risk of cardiovascular disease (CVD) later in life [2, 3]. Preeclampsia (PE), a hypertensive disorder of pregnancy characterized by new onset of hypertension in combination with proteinuria or end-organ dysfunction, affects approximately 2–8% of pregnancies [4, 5]. Beyond acute morbidity and mortality, PE is associated with a two-fold increased lifetime risk of ischemic heart disease, stroke, and cardiovascular death, and a four-fold increased risk of subsequent heart failure and hypertension [6]. The American Heart Association considers PE to be a CVD risk factor comparable to smoking and diabetes mellitus that could unmask early vascular or metabolic disease [7]. There is consistent evidence that adherence to healthy lifestyle behaviors, including healthy diet, physical activity, alcohol moderation, and smoking cessation can optimize modifiable cardiovascular risk factors and reduce the risk of CVD [8].

As part of routine postpartum care, it is usually recommended that women have a check-up scheduled 6–8 weeks after the birth [9]. International guidelines on postpartum follow-up of women with PE differ and there is no international consensus on whom to include, when to start or how to follow up [10, 11]. The most comprehensive clinical guidelines for postpartum follow-up after PE recommend up to yearly blood pressure monitoring and assessment of CVD risk factors [11]. Norwegian national guidelines on postpartum follow-up after PE recommend that women with PE should be informed of the increased cardiovascular risk and receive subsequent follow-up care with assessment of CVD risk factors [12]. However, postpartum risk counseling is often not routine among primary health care providers [13], and although women with PE are more likely to attend the recommended postpartum visit, the overall rates of follow-up are low [14].

In a scoping review from 2019, six out of seven studies found that women had limited to no knowledge on the increased risk of future CVD following a hypertensive pregnancy disorder [15]. The review revealed that even though the majority of health care providers were aware of the long-term risks and existing guidelines, follow-up care was still inadequate [15]. Another study found that women who were aware of the link between hypertensive disorders of pregnancy and CVD were significantly more likely to receive antihypertensive monitoring and treatment than their unaware peers [16]. This represents a substantial missed opportunity to provide postpartum counseling, screening and treatment to women at risk of developing short- and long-term cardiovascular sequelae. A qualitative study in the US using data from the Preeclampsia Foundation described how women called for improved postpartum counseling and management after PE [17]. Furthermore, qualitative studies in Norway and the Netherlands reported that women appreciated postpartum risk counseling and were motivated to make lifestyle changes [18, 19].

However, we lack knowledge about how women with PE experience current postpartum care in the Norwegian health system. Our aim is to explore the perspectives of women with a history of PE participating in a lifestyle intervention study, their risk perception, interaction with their health care providers, and their knowledge and attitudes regarding postpartum preventive care. A better understanding of how women with complicated pregnancies experience their postpartum care may help to improve the current postpartum management of PE and, in the longer run, to reduce the overall cardiovascular risk in women.

## Methods

### Study design

This study has a qualitative design, which is appropriate when the purpose is to obtain insight and understanding, such as when assessing personal experiences, understanding and interaction [20]. The research team consisted of two female medical students interested in the topic of women’s health but without previous experience in postpartum care (EA and RJ), an experienced midwife and doctoral student (HLS) with broad theoretical knowledge and practical experience in qualitative research, and an obstetrician (JH) with clinical experience in postpartum care, qualitative research experience and interest in the association of pregnancy complications and later maternal cardiovascular health.

The data used in this project were provided by ‘Mom’s Healthy Heart’ (MHH), a single arm lifestyle intervention study for women with recent PE or gestational diabetes [21]. This six-month lifestyle intervention program included phone-based counseling by a registered dietitian and access to the MHH website containing information on a healthy diet, physical activity and motivation for lifestyle changes. MHH focused not primarily on weight reduction but on increasing adherence with the Norwegian food-based dietary guidelines and physical activity [22]. MHH collected data through questionnaires and clinical measurements at baseline, and at three- and six-month follow-up study visits. Further, all participants were invited to a semi-structured telephone interview after completing the intervention program.

Potentially eligible participants in the MHH study were women aged 18 years or older with a recent pregnancy complicated by PE and/or gestational diabetes resulting in a live birth 3–12 months prior to recruitment at Levanger Hospital or St. Olavs University Hospital, two hospitals in central Norway. Potential participants were identified by the electronic patient administrative system of the two hospitals. For practical reasons, eligible women had to live at most two hours’ drive from one of the two hospitals. Diagnoses of PE and gestational diabetes were validated according to international diagnostic criteria based on their medical records [3, 6]. A diagnosis of PE required de novo hypertension after 20 weeks of gestation in combination with proteinuria or with other signs of organ dysfunction. Severe PE was characterized by severe hypertension (blood pressure ≥ 160 mmHg systolic or ≥ 110 mmHg diastolic) or PE with signs of significant end-organ dysfunction [12].

Participants were recruited by mail and interested women returned a signed consent form. After this, the last author (JH) or a registered dietitian contacted potential participants by phone, explained the study, answered questions, and assessed exclusion criteria. These included the inability to speak and read Norwegian, current pregnancy, diagnosis of chronic hypertension, diabetes mellitus or hypercholesterolemia, current use of medication to lower blood pressure or cholesterol, diagnosis of eating disorder, heart disease, stroke, or kidney disease, and previous gastric bypass surgery. Forty-four women were included in the study, and forty (17 preeclampsia, 23 gestational diabetes) completed the six-month intervention program.

### Participants

Our study population comprised 17 women with a recent history of PE who had completed a 6-month postpartum lifestyle intervention. They were 9–20 months postpartum when participating in the semi-structured interview as a final assessment of the MHH study. Table 1 provides information on demographic and pregnancy characteristics. The participants varied in age, educational level and household income. The majority were Norwegian, two reported other European nationalities and most women were primiparous. All women were married or cohabiting. Half of the participants had experienced severe PE and preterm birth.

**Table 1:** Participant demographics and pregnancy characteristics (*n* = 17)

Characteristics	n (%)
**Age (years)**	
< 30	9 (53)
30–34	5 (29)
≥ 35	3 (18)
**Ethnicity**	
Norwegian	15 (88)
Other European	2 (12)
**Marital status**	
Married	3 (18)
Cohabiting	14 (82)
**Education**	
Secondary education	4 (24)
Lower tertiary education (< 4 years)	6 (35)
Upper tertiary education (≥ 4 years)	7 (41)
**Household income**	
< 450,000 NOK	2 (12)
450,000 – 1,000,000 NOK	7 (41)
> 1,000,000 NOK	8 (47)
**Parity**	
Primiparous	15 (88)
Multiparous	2 (12)
**Gestational age**	
< 37 weeks	8 (47)
≥ 37 weeks	9 (53)
**Time since delivery**	
< 12 months	3 (18)
≥ 12 months	14 (82)
**Severity of preeclampsia**	
Moderate	8 (47)
Severe	9 (53)
**Smoking**^a^	
Never	13 (76)
Former	4 (24)
Current	0 (0)
**Body mass index**^a^	
< 25 kg/m^2^	4 (24)
25- < 30 kg/m^2^	7 (41)
≥ 30 kg/m^2^	6 (35)

^a^At baseline after recruitment in MHH

### Data collection

Interviews were conducted by telephone by the last author (JH) and audiotaped between January and October 2021. Although more direct forms of communication would normally have been preferable, face-to-face interviews were not possible due to national COVID-19 restrictions, and some cases of women living remotely. The interviewer was the principal investigator of the MHH study and had short prior contact to some of the participants under the recruitment process to answer questions about the study and assess exclusion criteria. Ranging from 15 to 70 min duration (average of 34 min), the interviews were conducted individually (one-on-one) and followed a semi-structured format based on a predefined interview guide (Additional file 1). The interview guide was developed specifically for MHH to explore the participants’ experiences with the intervention study as well as with their postpartum follow-up (apart from participation in MHH). Three researchers contributed to the development of the interview guide based on their experience and prior literature: the last author (JH), a registered dietitian with experience in postpartum lifestyle counseling and a qualitative researcher with background in intensive care nursing. Socio-demographic data were collected using a questionnaire at baseline.

Women in the study were offered a choice of several dates and times for the interview. They were reminded that the interview was voluntary, informed about privacy protection, and told that they could skip any question. In the interview, they were asked to detail their experiences of interaction with different health care providers during their postpartum follow-up. Further, the interviewers explored their perceptions of their CVD risk, postpartum screening and preventive care.

### Data analysis

Malterud’s systematic text condensation, a four-step analysis method based on a thematic understanding [20], was used in the data analysis. Systematic text condensation is inspired by Giorgi’s phenomenological approach and aims for meaning and content of data across cases [20]. First, the interviews were transcribed verbatim. Transcripts were then read several times by the first authors (EA and RJ) simultaneously, who made reflective notes to become familiar with the data and explore preliminary themes associated with the participants’ experiences of postpartum follow-up [20].

Step two involved developing preliminary code groups with underlying subgroups. Corresponding meaning units were further sorted into these code groups. The research group resolved discrepancies found in step one through reflective discussion until consensus was reached. By identifying and sorting meaning units that were potentially related to our new themes, we worked our way through the coding. The codes were developed continuously as the analysis led to ideas and suggestions [20].

In step three, the empirical data were reduced to a decontextualized selection of meaning units sorted as thematic code groups across individual participants [20]. Condensates were created from the meaning units to form a coherent text that embodied the content of the interviews. The analysis process was iterative, and we made an ongoing effort to capture information that had previously been missed as the code groups evolved. Although data saturation was evident around interview number 12, we decided to continue to explore data as planned from all 17 participants to ensure that no new codes or themes were identified.

In the fourth step of the analysis, the data were re-conceptualized, i.e., the pieces were put back together. Our end goal was to provide credible stories that could make a difference by elucidating the topic under study [20]. The condensates were converted into analytic texts by carefully retaining the participants’ voices, while describing all the content from an outsider’s point of view.

Consolidated criteria for reporting qualitative studies (COREQ) 32-item checklist was used for reporting [23].

### Ethics

Ethical approval for the Mom’s Healthy Heart study, including this qualitative study, was obtained from the Central Norway Regional Committee for Medical Research Ethics (REK Central, 2018/1803). All participants provided written informed consent prior to inclusion. In addition, verbal consent to audio record the conversation was obtained before each interview. Transcripts were anonymized using pseudonyms.

## Results

We identified five themes, each with 2–3 subthemes, that described how participants with recent PE experience postpartum follow-up: (1) fear and uncertainty, (2) a conversation on lifestyle – not really that difficult, (3) when your own health is not a priority, (4) motivation for lifestyle changes, and (5) lack of structured and organized follow-up.

### Fear and uncertainty

#### A body out of balance

Most participants in this study were unprepared for the challenges that awaited them after a pregnancy complicated by PE. A few women wondered why they in particular developed this complication. They had no history of health problems and associated hypertensive disorders with older people. Some participants even began to wonder if they were to blame for getting PE. Furthermore, they explained that it was difficult to process information on risk factors and causes of PE given to them by health professionals. It was challenging to process any input while trying to recover from a complicated pregnancy and simultaneously being responsible for a newborn baby. One participant summed up her thoughts well:

*“I don’t think you can absorb that information until you’re ready for it. And I don’t think you’re ready until you’ve given birth, recovered from preeclampsia, and some time has passed. Because you’re in quite a state of shock afterwards. I was at least. I was shocked at how things could go so fast and so wrong.”* (Participant 10)

Several participants emphasized that external stress and hormonal changes contributed to their overall strain. They also pointed out how their needs for support and reassurance were often met by family members or partners, not by health professionals as some had expected to a greater extent.

#### Facing an uncertain future

Many participants were anxious about how PE might impact their future health. They wondered what the condition implied and what awaited them in the future. A few sought clarification from health care professionals without receiving any helpful answers. The hospital never confirmed that they in fact had suffered from PE. A number of participants were hospitalized for several days, some even for weeks. Despite this, there was little talk of what had happened. Some of the participants were rebuffed when they tried to reach out to health care professionals.

In different ways, many participants expressed a feeling of neglect. One participant recalled thinking: *“I did not feel prioritized. I was just lying there in uncertainty.”* (Participant 11).

The feeling of abandonment persisted even after discharge from hospital. Some stated that they would never get pregnant again without having their own blood pressure monitor. Others mentioned how they used Google to learn about their need for blood thinners in any future pregnancies. Some still wondered what might happen if they developed high blood pressure again. A common perception among these new mothers was that their concerns were not taken seriously and properly followed up. Some participants expressed concern that health care professionals withheld information about the causes and consequences of PE. *“I was upset that no one had a conversation with me to explain what it really means and why I got preeclampsia while other people don’t get it. And also what the consequences would be.”* (Participant 14).

The participants had varying previous health knowledge, personal coping resources and social support to help them get through their ordeal. Afterwards, they gave much of the credit for how things turned out well to their own resilience and their strong family support.

### A conversation on lifestyle – not really that difficult

#### Preeclampsia as a gateway

Several participants argued that lifestyle counselling should be mandatory after PE. Some participants believed that fear of offending patients was a common barrier for health care professionals when addressing weight and lifestyle. The participants shared this view regardless of whether they felt they had a healthy lifestyle or were dissatisfied with it and wanted a change. A national checklist of topics that should be included in a conversation on lifestyle was suggested. One of the participants put it this way:

*“It should be mandatory. Just as natural as taking your baby to the health center, it should be a natural part of becoming a mother with the challenges that obesity presents. That way, you would also be prepared for that conversation, and perhaps it would be easier to talk about lifestyle with health care staff.”* (Participant 6)

On the other hand, some participants pointed out that they did not have a weight problem and therefore did not need a conversation on lifestyle. One participant stated that it would be more natural to discuss lifestyle and health if it was a mandatory topic of conversation after PE. PE as a gateway to talking about lifestyle changes could perhaps make it a more accessible topic.

#### A respectful approach

Many participants emphasized how a respectful approach was important for them to feel that lifestyle counselling by their healthcare provider was constructive. An open and friendly dialog was highlighted by several participants as more crucial to their experience of the conversation than the content or topic itself. One participant emphasized that health care professionals should have an objective, advisory role. After all, they already talked to their patients about other serious and sensitive topics. Lifestyle counseling should not be an exception. Several participants clearly had different emotions related to weight and lifestyle. One participant put it this way: *“I think it’s difficult and sad that I didn’t succeed. I’m not proud of myself and I wish things were different. But it’s still okay to talk about it. I won’t be down in the dumps because of that.”* (Participant 6).

Several approaches that could help maintain the women’s self-esteem during such a dialog were presented. A common suggestion was to start with open and neutral questions to invite women to present their subjective understanding of their lifestyle. Their desire and willingness to change could then be identified. The participants emphasized how self-awareness and willingness to change were crucial to utilize the help offered by health care professionals. One of them suggested the following:

*“Ask her carefully what she thinks of her lifestyle. Is it anything she would like to change? And is she aware that it can affect this or that? But my opinion is that if someone has a musculoskeletal injury due to obesity, you have to be a bit direct as well. Only then might she be able to think, ‘Hey, now I gotta do something’.”* (Participant 13)

#### A desire for more constructive feedback

Many longed for a more fruitful lifestyle conversation. They emphasized that it had to include more than just advice to change one’s diet and increase one’s physical activity. The purpose of the conversation should be to communicate facts and knowledge to the women. *“Health care professionals have a responsibility to raise people’s awareness about lifestyle and so on (…) I just don’t understand why they don’t bring it up.”* (Participant 16).

Several participants also wanted advice on how to organize exercise and on what a healthy diet consists of. Most of them understood that the aim of a lifestyle conversation was to help and motivate them, rather than to make them feel guilty. As long as a respectful approach was adopted, several participants wanted a more direct and honest way of communication. They also called for specific advice and long-term follow-up. One participant said:

*“I would have appreciated a conversation on lifestyle, but it would have to be more constructive than just instructions to exercise and eat healthy food (...). The fact that we have a weight problem comes as no surprise to us who struggle with overweight. Maybe we should think that we’re not afraid of that conversation, but not just to hear that we’re fat, but to be told about the help that’s available.”* (Participant 4)

### When your own health is not a priority

Participants described several external barriers to health promotion specific to early parenthood.

#### A new everyday life

Most participants found that their daily lives changed after giving birth. They had less time and energy to pursue a healthy lifestyle. Many fell back into old habits despite a strong desire to care for themselves and make healthy lifestyle choices. One of the participants explained it all very succinctly: *“It’s my everyday life that challenges what was supposed to be my healthy lifestyle.”* (Participant 5).

According to these participants, a busy life was often what prevented them from making lifestyle changes after PE. At times they found it difficult to get enough sleep. Caring for their baby came first, which, coupled with studies or a full-time job, compromised any plans to improve their health.

#### Out of focus

Just like any other new mother, the participants focused on the newborn baby during the postnatal period. Several stated that this also applied to the staff. Many participants found it natural to focus strongly on the baby, but later realized that things should have been different:

*“So there wasn’t much information and attention for me, and the fact that I’d had a major operation (...). Of course I think it was good to focus a lot on the baby, but it would have been nice to get some information about my health and my body and my reaction after that kind of experience.”* (Participant 13)

By the time the interview took place, a few months after the baby was born, the majority of the participants had come to the conclusion that they themselves needed more care following discharge. There were regular check-ups at the health center for the baby, but few or no check-ups for the mother. One participant pointed out that the appointments at the health center were for the baby, not the mother, which made it difficult to ask questions about her own health. She was left with many unanswered questions, as she had not seen a midwife or physician again since discharge.

#### Lack of support

The interviews revealed that the participants’ partner, family members and colleagues were not always supportive of their journey towards recovery and better health, and how the participant and the others involved were often not fully aware of this. Many participants felt an overriding responsibility for their household, which occupied much of their time. Some said that if they prioritized a healthy diet and refused cake during family visits, they were viewed as obstinate. A few told stories of how their partners could be very supportive in their words, but rarely in their actions, in terms of letting the participant get more “me-time”. When there were shortages of staff at work, many felt pressured to work overtime. *“Maybe I don’t prioritize myself because I get such a guilty conscience if I do.”* (Participant 6).

External pressure from several sources surrounding the new mothers resulted in a guilty conscience if they prioritized themselves and their own health over their children, partner, family, and work.

### Motivation for lifestyle changes

Although many participants experienced PE as a wake-up call for health promotion, they described social support as a critical factor in enhancing their intrinsic motivation.

#### An eye-opener

Having experienced PE was a wake-up call for many of the participants for how they perceived their health. After bringing a new life into the world, they also emphasized the long-term perspective on their health. Many expressed fear of the long-term consequences of PE and of getting PE in their next pregnancy. This often resulted in a strong desire to take preventive measures. *“There’s a lot of heart disease in my family. That’s a trigger to start with, and now getting preeclampsia too, so … now something has to change. Now I have to start taking care of myself.”* (Participant 10).

Several participants reported having received no information about long-term consequences or the association between PE and lifestyle. They therefore lacked knowledge of how to take preventive measures. Most wanted to receive information about the risks and long-term consequences after the postnatal period, while the dramatic experience was still fresh in their minds.

*“Right after the birth is a bit early, but the six-week check-up would have been a good time for me. It’s okay to address it while you’re still a bit affected by it, you shouldn’t wait too long. I think the information should be given early, especially if you want to change your diet and start being more active.”* (Participant 4)

#### Lack of intrinsic motivation

In order to achieve their goals for exercise and diet, several participants sought external support. Some even wanted others to be strict and tell them what to do. That would have made them accountable for their progress or lack of it. Others wanted a setting where they could learn about nutrition and physical activity. *“I want more knowledge about nutrition, it’s easy to be overwhelmed if you start on your own.”* (Participant 13).

Some participants had previous experience of dieting and admitted that quick results were crucial for their motivation. Others pointed out how the weather and time played a major role in whether they prioritized exercise and diet. Some were motivated by partner involvement, while others found it demotivating that their partner was in better physical condition than they were themselves.

#### A helping hand

Support from others was seen as a crucial factor for maintaining lifestyle changes. Several participants found it helpful to talk to others about diet and exercise, both professionals and women with similar experiences. Many strongly emphasized the value of having someone to support and motivate them. In that context, several mentioned how they missed an organized lifestyle initiative targeting women who had had PE. Some also wanted their partner to be involved in any initiative, as they felt alone in their desire to make healthy choices. *“After all, my partner and I eat the same food and go for walks together. It would be easier if we were both making healthy choices.”* (Participant 9).

Several participants felt that lifestyle change was mainly about finding the will to change and being aware of various choices related to food or activities. Some already had an interest in health and exercise and felt that their lifestyle was healthy. However, despite many attempts, others had been unable to make changes on their own. One participant said: *“I’ve been trying for 10 or 15 years to figure out what to do, but I still haven’t found the miracle cure to get a healthier life. What I find difficult is how to get there.”* (Participant 3).

### Lack of structured and organized follow-up

#### There should be a proper system

Many participants reported feeling well cared for when admitted to hospital, but found that things changed following discharge. They perceived follow-up care after PE as completely or partially inadequate. Those who considered themselves resourceful had contacted health professionals themselves in order to initiate follow-up care. They worried about what happened to the women who did not seek help on their own. *“Things at the hospital were great. Afterwards there was absolutely nothing.”* (Participant 1).

Several participants reflected on what could have been done differently to ensure that no one was neglected by the health care system. One participant made the following statement: *“The follow-up should have been like the cervical screening program, where you get a reminder. There should a proper system.”* (Participant 13).

#### A one-sided focus in follow-up care

Each participant had a personal story about her post-pregnancy follow-up experience. The follow-up care they received had varied considerably. Some felt lucky to have had a good conversation about the birth, future contraception and blood pressure monitoring for the next year. Others had not even been to their physician for a postnatal check-up. Most of the participants knew that a six-week check-up existed, but were not sure about whether or not they actually had attended one. The majority had their blood pressure checked during a consultation, but this was not followed up for most of them. Some had their abdomen checked, while others were only asked if everything was fine. Thus, their experiences of follow-up care or the lack of it varied considerably.

Three participants told us what they felt was missing: *“My doctor checked my blood pressure, but he never brought up anything else. Nothing about my preeclampsia or anything else about the birth.”* (Participant 16)*, “The only thing my doctor told me was information from my hospital discharge letter. Nothing about diet or exercising.”* (Participant 13) and *“My doctor forgot about what’s supposed to be basic blood tests after pregnancy.”* (Participant 10).

After a challenging pregnancy complicated by PE, these participants missed a more nuanced first consultation after giving birth, preferably with more focus on lifestyle changes and their future health.

#### Individual variation in follow-up care

Despite the broad consensus among participants about various inadequacies in their follow-up, some of the participants were very positive about the care they had received. Some participants felt very cared for and seen by their primary care physician, as the physician had addressed their hypertension and continued to monitor it for a long time. In addition, they spoke of good conversations about how their birth had actually been. Others told stories of proactive midwives who were the ones who discovered how these participants were severely ill after discharge from hospital and quickly referred them directly to the hospital or their physician. *“I also had a conversation at the hospital. One of those debriefings. And that was a very nice conversation where I got to ask about everything I was wondering about. And how to go about things if we want more children in the future.”* (Participant 17).

When the participants were asked directly about whom they would have preferred to be in charge of their follow-up care, their answers varied. The majority said either their primary care physician or a midwife, while a few participants considered a specialist in obstetrics or gynecology to be more suitable. However, the participants’ main point was that someone should take responsibility for follow-up care. *“Either the midwife or the doctor could do the follow-up, but one of them should have the main responsibility, so they know who’s actually doing it.”* (Participant 4).

## Discussion

This qualitative study identified multiple factors that can impact postpartum follow-up of women with PE. Lack of knowledge on the association between PE and future CVD risk, poor transition from obstetric to primary care, inconsistency of follow-up, and a lack of support for lifestyle changes are factors contributing to missed opportunities for CVD prevention.

Prior research on postpartum care after PE has suggested a fragmented health care system and poor obstetric-primary care transition as barriers to the initiation of health behavior changes in the postpartum period [24]. The women in our study likewise described a contrast between close antenatal monitoring and little contact with health care providers postpartum. Although both antenatal and postpartum care in Norway are mainly provided by midwives and physicians in primary health care, sub-optimal interprofessional collaboration and a lack of knowledge of the association between PE and CVD may affect the transition of care [25]. One potential reason for fragmentation in Norwegian primary health care is the role distribution, where antenatal care is commonly provided by midwives, while primary care physicians mostly deliver postpartum follow-up care. Other researchers have found poor knowledge of the link between PE and cardiovascular risk among health care providers, especially internists and primary care physicians [24, 26]. They suggested that this might be attributed to the low prevalence setting of primary care [24].

In line with previous studies, our findings suggest that a routine invitation to postpartum follow-up, similar to the invitation to cervical cancer screenings, could encourage participation of women with recent pregnancy complications [25, 27]. Furthermore, discharge information written for women with PE discharged from maternity wards could improve their understanding of their medical condition and their ability to ask questions and make informed choices regarding their postpartum health care.

Despite existing guidelines for postpartum follow-up after PE, women in our study often found postpartum care to be inconsistent and unsystematic. This is in accordance with previous studies from Germany, the US, and India, which indicated suboptimal adherence to clinical guidelines for postpartum follow-up after PE [28–30]. Simplified checklists for postpartum care providers, such as those developed by Morgan et al. [31], could provide the structure which is lacking today. It is recommended that lifestyle and CVD should be addressed as part of the postpartum follow-up, as PE is considered a manifestation of cardiovascular vulnerability [13, 32]. However, as in previous studies, we found that even motivated women did not receive advice about lifestyle change during postpartum visits [24, 33, 34]. Both the women and the health care professionals might find this conversation challenging, and including lifestyle advice in routine postpartum care could help to reduce these barriers in the long run [35–37]. The lack of intrinsic motivation for lifestyle changes that the participants in our study described suggest that the use of counseling strategies such as motivational interviewing might be important to support individuals to implement healthy lifestyle changes [38]. Similar to previous work on lifestyle behavior intervention adherence in adult populations in general [39], our study highlights the importance of social support for behavior change. This may be especially important for women after adverse pregnancy outcomes given the additional challenges in early parenthood.

In our study, neither the women themselves nor their health care providers focused on maternal health postpartum. As maternal lifestyle is strongly associated with the risk of obesity in offspring [40, 41], this represents a wasted opportunity not only to improve maternal health but also to interrupt a generational downward spiral. We therefore agree with other researchers who have suggested that lifestyle interventions postpartum should take a family-focused approach due to the crucial role of partner and family support for lifestyle changes [42, 43].

### Study strengths and limitations

To enhance validity, the research process is clearly described and the study findings are illustrated with original quotes. Most study participants were born and raised in Norway and reported a high income and educational level. However, our sample showed variation in socioeconomic status, place of residence, parity, and severity of PE. It is important to note that participants were recruited from an intervention study for women with recent PE and had completed a lifestyle intervention program. Standard postpartum care in Norway includes a postpartum contact with a midwife or primary care provider 6–8 weeks after delivery. Participants entered the intervention study 3–12 months after delivery and study participation is unlikely to have altered their standard postpartum care. However, participants were probably more motivated for lifestyle changes than women with recent PE in general and their views on postpartum care may therefore differ from those of other women with a history of PE. Although the use of telephone interviews instead of face-to-face interviews resulted in a lack of non-verbal communication, conducting phone interviews provided greater flexibility for participants and may also have reduced visual biases and socially desirable responses [44]. There was a strong commitment from the research group to maintain reflective notes and regularly discuss data analysis and interpretation to limit personal biases. However, the research group has a strong background in obstetrics and the inclusion of primary health care researchers may have enabled us to view the participants’ experiences within a broader perspective.

## Conclusions

Findings from this study highlight the need for more systematic postpartum follow-up care for women after a pregnancy complicated by PE. Women with other adverse pregnancy outcomes associated with increased risk for cardiovascular disease would presumably also benefit from a greater focus on preventive postpartum healthcare [3]. Further research is required to explore the potential use of standardized guidelines and routine invitations to postpartum care. Furthermore, exploring health care professionals’ experiences is crucial to ensure their engagement in postpartum care following complicated pregnancies.

### Supplementary Information

**Additional file 1.**

## Data Availability

The interviews transcribed for the present study are not publicly available due to individual privacy considerations. All data requests should be submitted to the corresponding author for consideration. Access to anonymized data may be granted upon reasonable request, subject to approval by the Central Norway Regional Committee for Medical and Health Research Ethics.

## Abbreviations

CVD: Cardiovascular disease
MHH: Mom’s Healthy Heart
PE: Preeclampsia
